# Integrative GC–MS, FT‐IR, and Molecular Docking Analysis of *Drimycarpus racemosus* (Roxb.) Hook.f ex Marchand: In Vivo Analgesic and Antidiabetic Potential

**DOI:** 10.1002/fsn3.72204

**Published:** 2026-08-02

**Authors:** Md Sajib Ali, Md. Rakibul Islam, M. A. Rafi, Sanjana Haque Esha, Md. Mehedi Hasan, Payar Hossain, Abdullah Ripon, Nilufar Sultana

**Affiliations:** ^1^ Department of Pharmacy Manarat International University Dhaka Bangladesh; ^2^ Department of Pharmacy, Faculty of Biological Science University of Chittagong Chattogram Bangladesh

**Keywords:** analgesic, *Drimycarpus racemosus*, FT‐IR, GC–MS, hypoglycemic, molecular docking

## Abstract

Traditional medicine has long utilized *Drimycarpus racemosus* (Roxb.) Hook.f ex Marchand, which is a member of the Anacardiaceae family, to treat inflammation, pain, and metabolic disorders; however, its therapeutic properties have not been experimentally investigated. This study aimed to investigate the analgesic and hypoglycemic activities of *D. racemosus* leaf extract and explore its bioactive constituents through a computational approach. The most prevalent chemicals found in the plant methanolic extract, according to GC–MS analysis, were Furfural, 4‐methoxybenzoic acid, methyl ester and *β*‐sitosterol acetate. The FT‐IR (Fourier transform infrared spectroscopy) analysis of the extracts revealed the presence of hydroxyl, carbonyl, aromatic, aliphatic C–H, and C–O functional groups. Acetic acid‐induced writhing test, formalin‐induced hind paw licking test, and hot plate tests were used to assess the analgesic activity using Swiss albino mice, whereas streptozotocin (STZ)‐induced animals were used to assess the hypoglycemic activity. In silico docking experiments were performed using Gaussian, Gabedit, Discovery Studio, Swiss PDB Viewer, PyRx, and PyMOL tools against cyclooxygenase‐2 (COX‐2) and sulfonylurea receptor‐1 (SUR‐1), with ADME data supporting the results. The extract reduced writhing by 55.48% and 66.45% at 250 and 500 mg/kg, respectively. At 500 mg/kg, the extract significantly reduced paw licking time to 52.4 ± 5.95 s during the early phase and 3.4 ± 0.51 s during the late phase. At 500 mg/kg, a decrease in plasma glucose indicated hypoglycemic action. *In silico,* 4‐methoxybenzoic acid, methyl ester showed the strongest affinity for COX‐2 (−8.3 kcal/mol), outperforming diclofenac (−6.4 kcal/mol), while 4‐methoxybenzoic acid, methyl ester demonstrated higher binding with SUR‐1 (−9.0 kcal/mol) compared to Glibenclamide (−8.5 kcal/mol). *D. racemosus* extract has substantial analgesic and hypoglycemic properties, and its phytoconstituents show promise as new medicinal research possibilities.

## Graphical abstract

GC–MS profiling of *D. racemosus* identified bioactive phytochemicals with promising molecular interactions through docking analysis. The methanolic extract demonstrated significant antinociceptive activity against acetic acid‐ and formalin‐induced pain and exhibited antidiabetic effects in streptozotocin‐induced diabetic Swiss albino mice, supporting its therapeutic potential.

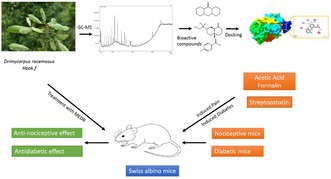

## 1 Introduction

Diabetes mellitus (DM) is a collection of complex illnesses characterized by persistently high blood glucose levels, or hyperglycemia (Astrup 2001). The International Diabetes Federation (IDF) indicates that approximately 589 million adults worldwide are living with diabetes as of 2025. While the projected increase to 693 million by 2045 appears to be from older data, more recent projections by the IDF suggest the number could rise to 853 million by 2050 (Magliano and Boyko 2025). The primary result of diabetes mellitus is high blood sugar, which is brought on by a lack of insulin production as well as an aberration in the beta cells in the pancreas that produce insulin (Gao et al. 2016). Diabetes‐related chronic hyperglycemia can cause long‐term damage, compromised function, and ultimately organ failure in several systems, including the blood vessels, heart, renal system, retina, and nervous system. Inhibiting the activity of enzymes involved in the digestion of carbohydrates, such as α‐glucosidase and α‐amylase, is one way to deal with this (Thilagam et al. 2013). Even though antidiabetic medicines from the groups of thiazolidinediones, α‐glucosidase, sulfonylureas, and *β*‐amylase inhibitors are used in combination or alone as medication for diabetes mellitus, these medications do have certain negative side effects (El‐Nashar et al. 2021; El‐Nashar et al. 2024). Around the world, 21,000 plants are widely used for their medicinal purposes due to their therapeutic properties, according to the World Health Organization (WHO). Of these, an increasing number of bioactive compounds derived from plant‐based sources have been found to have some potential for treating diabetes (Rizvi and Mishra 2013). The physiological and psychological aspects of pain in mammals are complex (Walters and Williams 2019). Pain is a subjective, disagreeable sensory and affective experience linked to present or prospective tissue damage and expressed in terms of that harm (Raja et al. 2020). The most commonly prescribed painkillers are non‐steroidal anti‐inflammatory drugs (NSAIDs), which are particularly non‐selective cyclooxygenase‐2 (COX‐2) inhibitors, and can have major side effects if taken excessively or regularly (Becker et al. 2004). Among the frequent worries are blood coagulation issues, stomach ulcers, hypersensitivity reactions, internal organ damage, and cardiac issues (Ho et al. 2011). Unfavorable side effects can occur even with selective COX‐2 inhibitors. For example, long‐term usage of rofecoxib is linked to a greater risk of stroke or heart attack (Dieppe et al. 2004). Using particular plant parts or their combinations, the traditional medical system of Ayurveda has documented several therapeutic solutions for common disorders that are comparatively free of the side effects typically associated with mainstream treatments (Mradu et al. 2013). One of the most understudied species in the commercially and medicinally significant Anacardiaceae family is *D. racemosus* . Although reliable botanical databases provide fundamental taxonomic and distributional information, the traditional use of the plant is unknown and the plant is lacking in thorough studies in almost every other field, such as phytochemistry, pharmacology, ethnobotany, ecology, and conservation biology (Kew Org 1869; Beaman 1986). The species has a great deal of potential for finding new chemical entities and biological activities because of its distribution throughout the biodiverse Indo‐Malayan area and its membership in a family known for bioactive compounds. But to fulfill this promise, methodical, interdisciplinary research projects that fill up the identified knowledge gaps are needed (Alamgir 2018). This study's ultimate goal is to evaluate the analgesic and hypoglycemic potential of the methanolic extract of *D. racemosus* leaves using both in vivo and in silico methods. Two therapeutic molecular targets that are antagonistic to cyclooxygenase‐2 (COX‐2) and sulfonylurea receptor‐1 (SUR‐1), respectively, were selected for this purpose.

## 2 Materials and Methods

### 2.1 Chemicals and Reagents

Methanol was acquired from Merck Germany, and the following ingredients were used: distilled water from Manarat International University, normal saline from Beximco Pharmaceuticals Ltd., and Streptozotocin (STZ) extrapure, 98% (CAS: 18883–66‐4) from Sisco Research Laboratories Pvt. Ltd. In this experiment, every chemical and reagent employed was of analytical grade.

### 2.2 Plant Collection

*D. racemosus* vernal leaves were collected in May 2025 from Ramgarh, Khagrachari. The Bangladesh National Herbarium in Mirpur, Dhaka, authenticated the plant (Accession Number: DACB124374). Scientific officer Khandakar Kamrul Islam of the Bangladesh National Herbarium (BNH) identified and verified the botanical identification.

### 2.3 Drying, Grinding, and Extraction of Leaves

**FIGURE 1:**
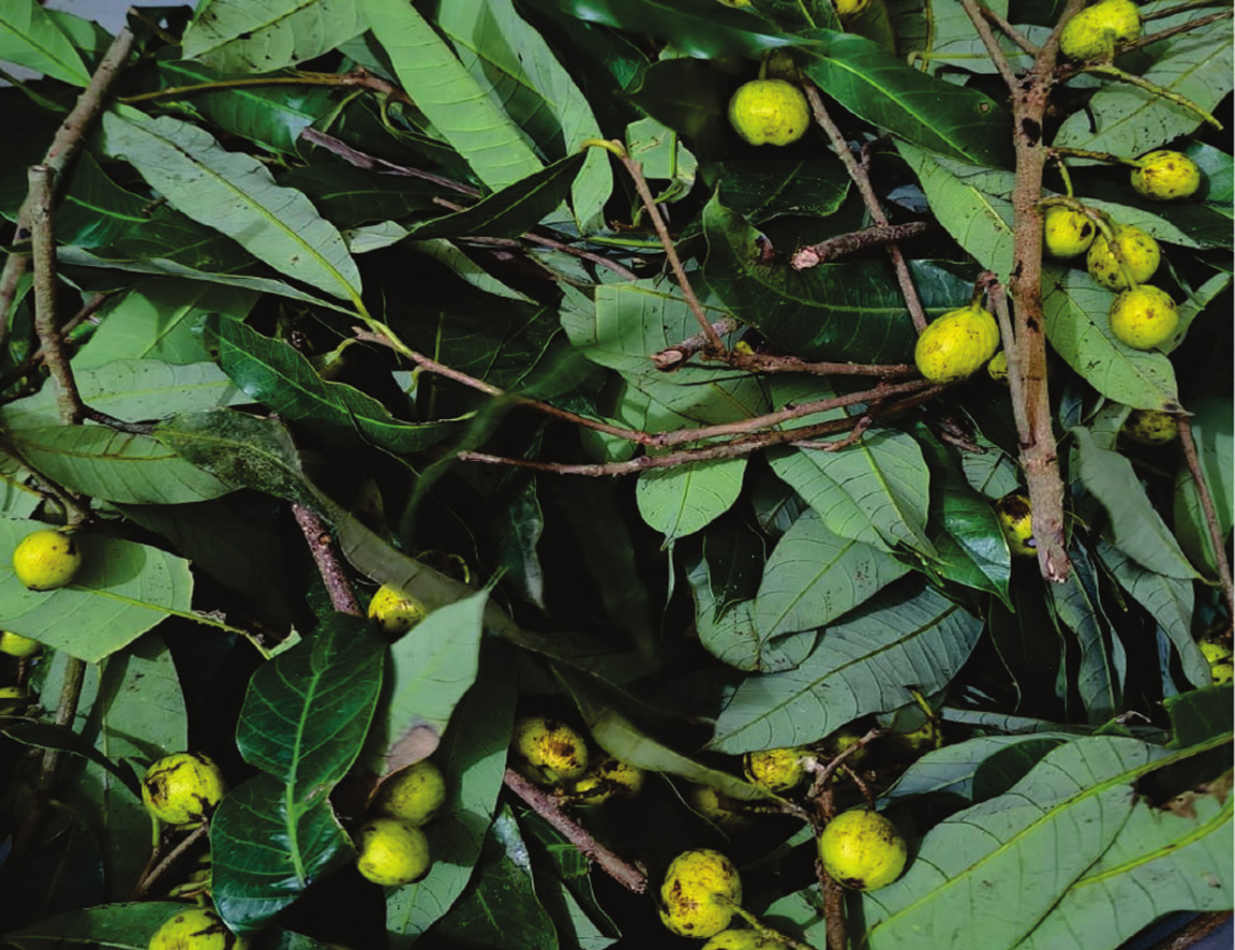
Collected leaves of *Drimycarpus racemosus* (Roxb.) Hook.f ex Marchand.

The leaves (See Figure 1) were shade‐dried at 27°C ± 2°C for 7 days and powdered using an electric grinder. Powdered materials were properly stored in a sealed bottle that offers protection against moisture infiltration. About 700 g of the powdered sample was macerated with 2.5 L of methanol for 10 days with occasional shaking at room temperature 25°C ± 2°C. Then the mixture was filtered to separate the liquid extract from the solid residues. After that, a white cotton cloth, free from dirt, marks, or stains, was used to sieve the blend of powder and solvent. The crude extract was obtained by evaporating methanol using a rotary evaporator at 40°C under reduced pressure. After evaporation of methanol, 35 g of crude extract was obtained from 700 g of powdered leaves, giving a yield of 5%. The yielded extract was transferred into a beaker with a proper seal. The crude extract was stored in a refrigerator at a temperature ranging from 2°C to 8°C.

### 2.4 In Vivo Studies

#### 2.4.1 Experimental Animals

Healthy Swiss albino mice of both sexes, aged 5–8 weeks and weighing 35–40 g, were procured from the Department of Pharmacy, Jahangirnagar University. Animals were randomly assigned to experimental groups, with each group consisting of 5 animals for analgesic study and 4 animals for hypoglycaemic study. Mice were housed in standard laboratory conditions with a temperature of 27°C ± 2°C, relative humidity of 55% ± 5%, and a 12 h light/dark cycle, with free access to food and water. All experimental procedures, including dosing and observations, were performed in accordance with ethical guidelines to minimize animal suffering and were approved by the Ethical Committee for Animal Studies at Manarat International University (Ethical code: MIU/SEST/ERC/2025/001). The study was designed, conducted, and reported in accordance with the ARRIVE 2.0 (Animal Research: Reporting of In Vivo Experiments) guidelines. Animals were randomly allocated to experimental groups, and all procedures were performed according to the approved ethical protocol. Detailed information on group allocation, dosing regimen, and observation is provided in the relevant sections.

### 2.5 GC–MS Analysis

A GCMS‐TQ8040 system (GC–MS/MS, Shimadzu) equipped with a mass spectrometer and a silica capillary column (DB‐5 ms, 30 m, 0.25 mm ID, 0.25 μm df) containing 95% dimethyl‐poly‐siloxane and 5% phenyl was used to examine the chemical components in plant extract following previously established GC–MS analytical protocols (Naz et al. 2014). Before analysis, the crude extract was dissolved in analytical‐grade methanol at an appropriate concentration and filtered through a 0.22 μm syringe filter to remove particulate matter. A 1 μL aliquot of the prepared sample was injected into the GC–MS system in split mode. Helium (99.99% purity) was used as the carrier gas at a constant flow rate of 1.0 mL/min. It ran for a total of 40 min. The material was analyzed using electron ionization at high energy (70 eV). The temperature of the column oven increased from 40°C to 300°C at a rate of 5°C per minute, while the intake temperature remained constant at 250°C. After that, it was maintained at this temperature for 2 min. The mass range was 50,600 m/z, and the scan period was 1 s. No derivatization procedure was applied before GC–MS analysis. The National Institute of Standards and Technology (NIST) database was used to identify the sample chemicals.

### 2.6 FT‐IR Analysis

The dried methanolic extracts were subjected to FTIR analysis (Perkin Elmer‐Model RZX) under the IR region in the range of 400–4000 cm^−1^, and the associated functional groups were determined (Coates 2006).

### 2.7 Acute Toxicity Study

Thirty Swiss albino male mice were randomly divided into five groups. Each group consists of six animals according to the guidelines of the Organization for Economic Cooperation and Development (OECD) for testing chemicals, with some minor modifications (Sewell et al. 2024). Five different doses (250, 500, 1000, 2000, and 4000 mg/kg) were administered to each group through an oral stainless‐steel gavage needle. After dosing, clinical indices of toxicity and mortality were monitored individually for each animal within the first 30 min, then monitored daily for the next 7 days. Toxicity signs are referred to as general behavior, weight loss, respiratory pattern, cardiovascular signs, motor activities, reflexes, and changes in skin and fur texture.

### 2.8 In Silico Analysis

#### 2.8.1 Preparation of Ligand

The 3D structures of all bioactive chemicals from the NCBI PubChem compound database in SDF format; Gaussian software was used to draw some of the compounds. They were then stored in mol format. The most stable and lowest energy‐containing conformer was fixed and saved in PDB format using the Amber potential for molecular dynamics and conformational search using Gabedit (version 2.5.0) software.

#### 2.8.2 Selection and Preparation of Protein

For evaluating the analgesic and hypoglycemic activity, two target receptors were selected: human cyclooxygenase‐2 (PDB ID: 5KIR, CAS Number 162011–90‐7) and sulfonylurea receptor‐1 (PDB ID: 6JB3, CAS Number 135062–02‐1). These are powerful molecular mediators and regulators of analgesic action and hypoglycemia; the macromolecules were obtained from the RCSB protein data bank (https://www.rcsb.org/) at resolutions of 2.70 Å and 3.53 Å, respectively. Unexpected chains, heteroatoms, water molecules, and co‐crystallized ligands were eliminated by the Discovery Studio Visualizer 2021 program. The Swiss PDB viewer program (Version 4.1.0) was used to minimize energy and eliminate bad atom interactions.

#### 2.8.3 Molecular Docking

Finally, molecular docking was performed by using PyRx software (version 0.8), considering the protein as a macromolecule and the compound as a ligand, and maintaining grid box sizes of 78.7375, 62.0317, and 64.0293 Å (for 5KIR) and 77.0135, 61.0808, and 87.5516 Å (for 6JB3) along the X, Y, and Z directions, respectively.

### 2.9 Evaluation of Analgesic Activity

#### 2.9.1 Acetic Acid‐Induced Writhing Test

For the analgesic activity assay, we followed an acetic acid‐induced writhing method described by Koster et al. with a few modifications. This approach was followed for carrying out the writhing test (Koster et al. 1959). For evaluation of analgesic activity by the writhing test, a total of 20 mice were categorized into 4 groups, each with five animals (*n* = 5). Distilled water was given to group 1 as a negative control. Diclofenac sodium (100 mg/kg) was administered to group 2 as a positive control. Methanolic extract of *D. racemosus* (250 and 500 mg/kg) was given orally to groups 3 and 4, respectively. Every animal was administered a 0.6% (v/v) acetic acid intraperitoneal injection with a dose of 10 mL/kg body weight. During a 5‐min time frame, 15 min after acetic acid administration, the multitude of writhing behaviors with each animal was noted. In addition, each group's mean abdominal writhing was calculated.

The percent inhibition of the writhing impulse was calculated using the following formula: (%) = 100 × (Cn—Ct)/Cn, where Cn = Average number of contractions in animals in the negative control, and Ct = Average number of contractions in animals given diclofenac sodium and different concentrations of *D. racemosus* extracts.

#### 2.9.2 Formalin‐Induced Hind Paw Licking Test

To evaluate the analgesic activity of methanolic extracts of *D. racemosus* leaves. 20 μL of 2.5% formalin was administered into the planar surface of the left hind paw of mice 30 min after administration of the samples (Hunskaar and Hole 1987). For the experiment, we divided 20 mice into 4 groups, each having five mice. Group 1 was given only distilled water (10 mL/kg per body weight), group 2 was given diclofenac sodium (100 mg/kg body weight), and groups 3 and 4 were given methanolic extract (250 and 500 mg/kg, respectively). All samples were administered orally 30 min before starting the experiment. The analgesic activity was measured separately; the first 5 min after formalin administration is considered as the early phase (0–5 min), and the time between 15 and 30 min was considered as the late phase. The index of nociception time is defined as the time that mice spent during paw licking.

#### 2.9.3 Hot Plate Test

For the hot plate approach, 20 mice were placed among 4 groups: negative control (distilled water), positive control and standard (morphine, 10 mg/kg), test group (250 and 500 mg/kg), each with five mice. Mice's paws are extremely sensitive to temperatures of 55°C ± 0.5°C. The experimental animals were positioned on a hot plate that was maintained at a constant temperature of 55°C ± 0.5°C. To avoid paw injury, a 20‐s cutoff interval was observed (Toma et al. 2003).

### 2.10 Evaluation of Hypoglycemic Activity

Twenty mice were divided into five groups, and each group consisted of 4 mice as follows: Control group (distilled water), Diabetic control, and induced diabetic mice administered with a vehicle solution (distilled water). Positive control, diabetic mice treated with Glibenclamide (10 mg/kg). Diabetic mice were treated with MEDR 250 mg/kg and 500 mg/kg BW. All of the treatments were continued for 21 days, and fasting plasma glucose level was measured on the 1st, 4th, 7th, 10th, 14th, 17th, and 21st day, followed by the method described by Jahan et al., with a few modifications (Jahan et al. 2022b). The dose rationale for the positive control was determined based on previous research (Pandit et al. 2010).

To develop diabetes in mice, a single intraperitoneal injection of streptozotocin (STZ) dissolved in 0.1 M citrate buffer with a pH of 4.5 was administered at a dose of 60 mg/kg. The STZ injections took about one working day to complete. Mice were required to fast before receiving the STZ injection on the first day of the experiment. During the fasting phase, the citrate buffer was prepared. To prevent STZ decomposition, the STZ was prepared just before injection. This dosing regimen is widely recognized to induce a Type 1 diabetes‐like condition through selective destruction of pancreatic β‐cells.

Blood samples were taken throughout 21 days of therapy, and glucose levels were measured to check the onset of diabetes. Fortunately, there was no animal waste, and hyperglycemia was induced for all twenty mice. For research on early‐stage type 1 diabetes (T1DM) mechanisms or for screening early treatments, STZ‐injected mice are confirmed as suitable for study when they exhibit hyperglycemia (blood glucose levels over 150 mg/dL or 8.3 mmol/L) that is statistically significant compared to control mice. This confirms that the STZ injection successfully destroyed enough pancreatic beta cells to induce a diabetic state (Furman 2015). Before the administration of the methanolic extract, in all the groups, the blood glucose level was measured. Glucose oxidase‐peroxidase reactive strips and a glucometer (ACCU‐CHEK Active) were used to assess blood glucose levels immediately.

### 2.11 Statistical Analysis

The results were expressed as mean ± standard error of the mean (SEM). Statistical analysis was conducted using the Statistical Package for the Social Sciences (SPSS, version 16.0, IBM Corporation, New York, USA). Repeated measures ANOVA followed by an appropriate post hoc test was employed for group comparisons. Statistical significance was determined using the following criteria: *p < 0.05, notably statistically significant.

## 3 Results

### 3.1 *GC* –MS Profiling of *D. racemosus* Extract

**FIGURE 2:**
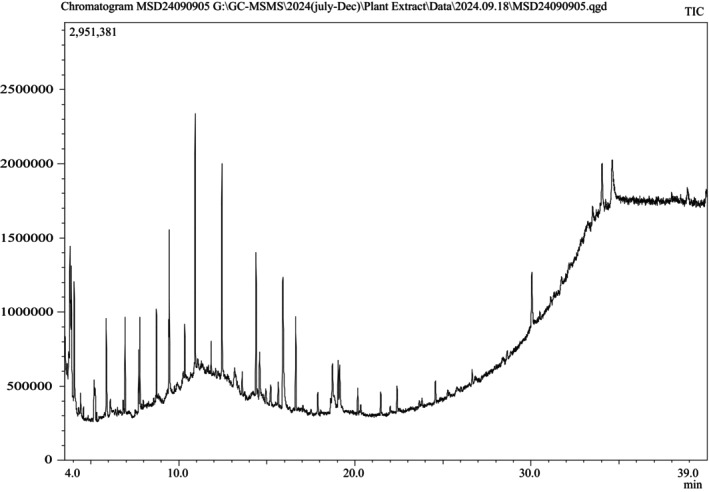
Chromatogram of methanolic *D. racemosus* extract through GC–MS analysis.

The GC–MS analysis of MEDR revealed roughly twenty‐seven bioactive components. The bioactive compounds are presented in Table 1, along with their names, relative retention times, and peak regions, including their chemical structures. The GC–MS yielded chromatogram has been presented in Figure 2.

**TABLE 1:** GC–MS yielded compounds of the methanolic *D. racemosus* extract leaves.

Sl. No.	R. time (minutes)	Area %	Compound name and molecular formula	Structure
1	3.53	4.25	4‐Nonenal, (E)‐ (C_9_H_16_O)	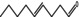
2	3.813	8.29	Furfural (C_5_H_4_O_2_)	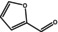
3	3.895	4.42	3‐Methylbenzyl alcohol (C_8_H_10_O)	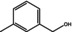
4	4.055	3.64	Retinal (C_20_H_28_O)	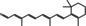
5	6.84	0.38	Vanillic Acid (C_8_H_8_O_4_)	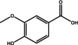
6	8.833	0.45	Naphthalenedione (C_10_H_6_O_2_)	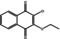
7	8.901	0.4	D‐Verbenone (C_10_H_14_O)	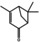
8	11.074	1.28	Tridecanoic acid, 4,8,12‐trimethyl‐, methyl ester (C_17_H_34_O_2_)	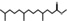
9	11.21	0.63	4‐Methylthiane (C_5_H_10_S)	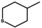
10	11.273	0.55	7‐Methyldecanoic acid (C_11_H_22_O_2_)	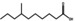
11	11.335	0.95	Methyl beta. ‐D‐glucopyranoside (C_7_H_14_O_6_)	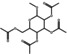
12	14.13	0.41	Laevo‐pinocarveol (C_10_H_16_O)	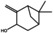
13	14.379	4	2,6‐Dihydroxybenzoic acid (C_7_H_6_O_4_)	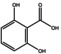
14	14.585	2.03	Neophytadiene (C_20_H_38_)	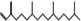
15	27.635	0.42	4‐Hydroxybenzoic acid (C_7_H_6_O_3_)	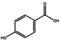
16	30.042	2.84	Squalene (C_30_H_50_)	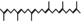
17	31.731	0.87	1,4‐naphthoquinone (C_10_H_6_O_2_)	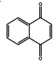
18	32.67	0.59	4‐methoxybenzoic acid (C_8_H_8_O_3_)	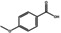
19	33.125	0.63	4‐methoxycinnamic acid, methyl ester (C_11_H_12_O_3_)	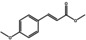
20	33.526	0.67	4 (15)‐Selinene‐11, 12‐diol (C_15_H_26_O_2_)	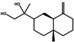
21	33.575	0.46	4‐methylbenzoic acid (C_8_H_8_O_2_)	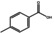
22	33.799	1	4‐methoxybenzoic acid, methyl ester (C_9_H_10_O_3_)	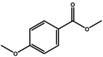
23	33.93	1.31	3‐methylsalicylic acid (C_8_H_8_O_3_)	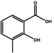
24	34.03	3.64	Beta‐sitosterol acetate (C_31_H_52_O_2_)	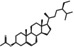
25	34.612	3.03	Alpha‐Tocopheryl acetate (C_31_H_52_O_3_)	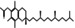
26	37.979	0.37	Propiophenone (C_9_H_10_O)	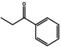
27	38.873	0.83	Feselol (C_15_H_26_O)	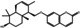

*Note:* Here, R. time means retention time.

### 3.2 FTIR Result

**FIGURE 3:**
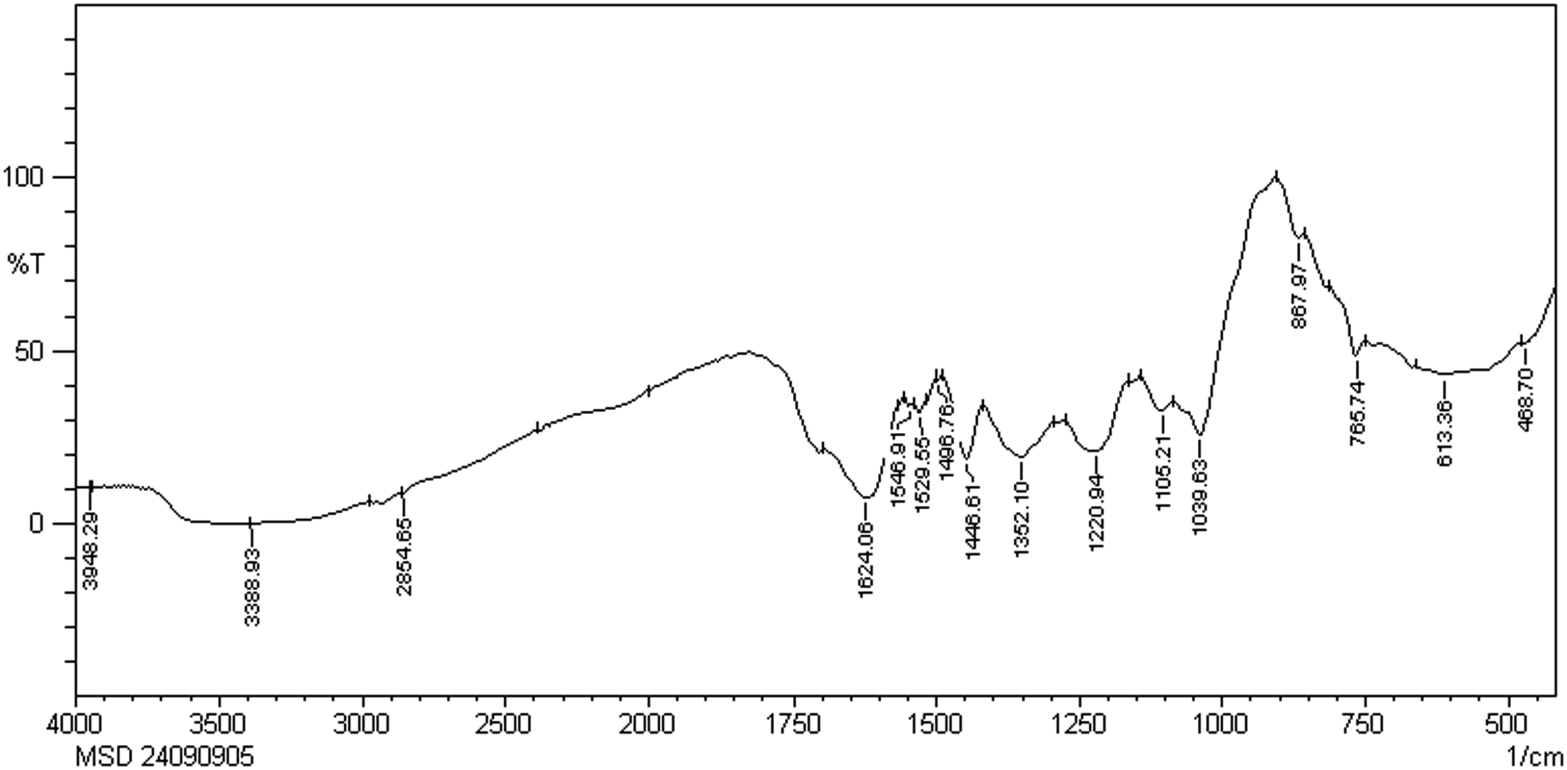
*FT‐IR spectra of* methanolic *D. racemosus* extract leaves.

FTIR has proven to be a valuable tool for the characterization and identification of compounds or functional groups (chemical bonds) present in an unknown mixture of plant extract (Sasidharan et al. 2010). The FTIR analysis of the samples was done, and the functional groups associated were determined. The IR spectrum of plant samples is shown in Figure 3. A broad band observed at 3408–3368 cm^−1^ was attributed to O–H stretching vibrations, indicating the presence of hydroxyl groups. The absorption band at 2948 cm^−1^ corresponded to aliphatic C–H stretching vibrations. A strong absorption at 1723 cm^−1^ was assigned to C=O stretching vibrations, suggesting the presence of a carbonyl functionality. The bands detected at 1659 cm^−1^ and 1584 cm^−1^ were associated with C=C stretching vibrations of aromatic or conjugated systems. The peaks at 1452 cm^−1^ and 1383 cm^−1^ were attributed to C–H bending vibrations of methyl and methylene groups. The absorption bands appearing at 1220 cm^−1^, 1103 cm^−1^, and 1018 cm^−1^ were assigned to C–O and C–O–C stretching vibrations, indicating the presence of alcohol, ether, or ester groups. Additionally, the band at 897 cm^−1^ corresponded to out‐of‐plane = C–H bending vibrations, while the peak at 766 cm^−1^ was characteristic of aromatic C–H out‐of‐plane bending, confirming substituted aromatic rings. The low‐frequency bands at 613 cm^−1^ and 486 cm^−1^ were associated with skeletal or halogen‐related vibrations. Overall, the FT‐IR spectral data confirm the presence of hydroxyl, carbonyl, aromatic, and C–O‐containing functional groups, which are commonly observed in phenolic and aromatic bioactive compounds.

### 3.3 In Vivo Result

#### 3.3.1 Acute Toxicity

There were no fatalities or abnormalities in the test animals at the experimental doses for each group, including restlessness, convulsions, decreased motor activity, diarrhea, coma, and lacrimation. As a result, LD50 was found to be greater than 4 g/kg of body weight.

### 3.4 Analgesic Test

#### 3.4.1 Acetic Acid‐Induced Writhing Test in Mice

**FIGURE 4:**
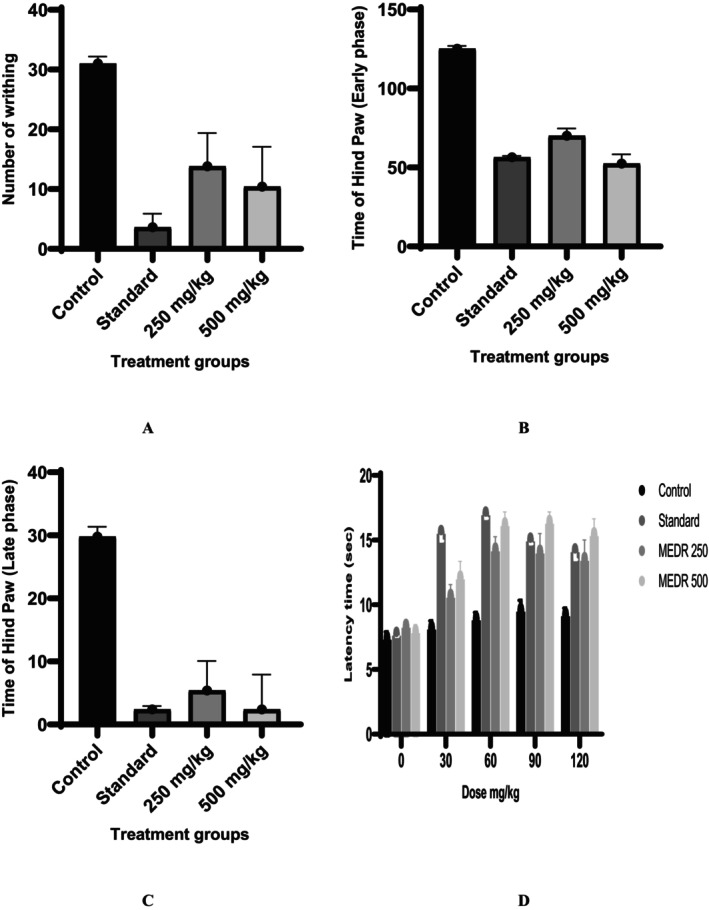
Analgesic effect of *Drimycarpus racemosus* leaves through (A) acetic acid‐induced writhing test, (B, C) Formalin‐induced hind paw licking test, (D) Hot plate test.

The effect of the MEDR leaves on acetic acid‐induced writhing in mice is given in Table 2. The control group had the most writhes (31 ± 1.18), indicating a typical pain response. The model's validity was confirmed by the standard drug, Diclofenac sodium (100 mg/kg), which significantly decreased writhing to 3.6 ± 2.28 with 88.39% inhibition. The number of writhes was reduced in a dose‐dependent manner by the MEDR. The extract produced 55.48% inhibition at 250 mg/kg and 66.45% inhibition at 500 mg/kg. See Figure 4. MEDR had a distinct analgesic effect, although it had a less potent impact than diclofenac sodium. The decrease in writhing implies that the extract could function by lowering the synthesis of chemicals that cause pain, including prostaglandins.

**TABLE 2:** Effects of MEDR in the acetic acid‐induced writhing test.

Animals group	Test sample	Dose	Number of writhing	Inhibition (%)
1	Control	10 mL/kg	31 ± 1.18	0
2	Diclofenac sodium	100 mg/kg	3.6 ± 2.28	88.39
3	MEDR	250 mg/kg	13.8 ± 5.58	55.48
4	MEDR	500 mg/kg	10.4 ± 6.68*	66.45

*Note:* Values were expressed as mean ± SEM (*𝑛* = 5). *p < 0.05 when compared with the control group (ANOVA followed by Dunnett's *𝑡*‐test).

#### 3.4.2 Formalin‐Induced Hind Paw Licking Test in Mice

The effect of the MEDR on formalin‐induced pain in mice is shown in Table 3. In the formalin‐induced paw licking test, the control group showed a mean licking time of 125.2 ± 1.71 s in the early phase and 29.8 ± 1.56 s in the late phase, indicating normal nociceptive and inflammatory responses. Diclofenac sodium (100 mg/kg) significantly reduced licking time to 56.4 ± 0.89 s in the early phase (54.95% inhibition) and 2.4 ± 0.51 s in the late phase (91.95% inhibition), confirming the effectiveness of the model. Treatment with the MEDR also produced significant, dose‐dependent analgesic effects. At 250 mg/kg, the extract inhibited paw licking by 44.09% in the early phase and 81.88% in the late phase, while at 500 mg/kg, the inhibition increased to 58.15% and 88.59%, respectively. See Figure 4.

**TABLE 3:** Effects of MEDR in the Formalin‐induced hind paw licking test.

Animal group	Test sample	Dose	Time (s) of Hind paw (Early Phase)	Inhibition % (Early Phase)	Time (s) of Hind paw (Late Phase)	Inhibition % (Late Phase)
1	Control	10 mL/kg	125.2 ± 1.71	0	29.8 ± 1.56	0
2	Diclofenac sodium	100 mg/kg	56.4 ± 0.89	54.95	2.4 ± 0.51	91.95
3	MEDR	250 mg/kg	70.0 ± 4.70	44.09	5.4 ± 0.46	81.88
4	MEDR	500 mg/kg	52.4 ± 5.95	58.15	3.4 ± 0.51*	88.59

*Note:* Values were expressed as mean ± SEM (*𝑛* = 5). *p < 0.05, when compared with the control group (ANOVA followed by Dunnett's *𝑡*‐test).

#### 3.4.3 Hot Plate Test in Mice

The effect of MEDR on the hot plate test in mice is shown in Table 4. Morphine (10 mg/kg), used as the standard drug, produced a marked and sustained increase in reaction time, peaking at 17.20 ± 0.53 s at 60 min, thereby confirming the validity of the model. Similarly, the MEDR significantly increased the reaction time in a dose and time‐dependent manner. At 250 mg/kg, the latency rose from 8.52 ± 0.28 s at 0 min to a maximum of 14.43 ± 0.82 s at 60 min, followed by a gradual decline, while at 500 mg/kg, the effect was more pronounced, with a peak latency of 16.55 ± 0.62 s at 90 min, approaching the analgesic activity of morphine. See Figure 4.

**TABLE 4:** Effects of MEDR in the hot plate test.

Animal group	Dose	0 min	30 min	60 min	90 min	120 min
Control	10 mL/kg	7.59 ± 0.32	8.38 ± 0.38	9.09 ± 0.32	9.75 ± 0.59	9.40 ± 0.34
Morphine	10 mg/kg	7.91 ± 0.36	15.78 ± 0.85	17.20 ± 0.53	15.19 ± 0.48	14.36 ± 0.87
MEDR	250 mg/kg	8.52 ± 0.28	10.84 ± 0.71	14.43 ± 0.82	14.26 ± 1.25	13.69 ± 1.32*
MEDR	500 mg/kg	8.11 ± 0.37	12.25 ± 1.11	16.37 ± 0.81	16.55 ± 0.62	15.61 ± 1.03*

*Note:* Values were expressed as mean ± SEM (*𝑛* = 5). *p < 0.05 when compared with the control group (ANOVA followed by Dunnett's *𝑡*‐test).

#### 3.4.4 Hypoglycemic Test in Mice

The effect of the extract of *D. racemosus* on the streptozotocin‐induced antidiabetic test in mice is shown in Table 5. In the hypoglycemic test, the control group showed no significant change in blood glucose levels over the 21 days, maintaining values close to baseline. In contrast, Glibenclamide (10 mg/kg) produced a marked increase in blood glucose reduction from day 4 onwards, reaching a peak on day 4 (285.84 ± 9.83 mg/dL) before gradually declining towards day 21 (146.52 ± 10.29 mg/dL), confirming the validity of the experimental model. Treatment with the MEDR also demonstrated significant and dose‐dependent hypoglycemic activity. At 250 mg/kg, blood glucose levels decreased progressively from day 4 (288.36 ± 11.59 mg/dL) to (87.12 ± 6.65 mg/dL) on day 21. Similarly, at 500 mg/kg, MEDR produced an even stronger reduction, with glucose levels falling from (256.68 ± 27.63 mg/dL) on day 4 to (83.52 ± 7.86 mg/dL) on day 21.

**TABLE 5:** Effects of MEDR in the hypoglycemic test.

Group	Dose	Initial	Day 1	Day 4	Day 7
Control	10 mL/kg	107.64 ± 7.19	106.2 ± 6.71	94.68 ± 4.50	109.08 ± 5.08
Diabetic Control	10 mg/kg	101.52 ± 4.98	100.44 ± 4.78	302.04 ± 7.36	327.60 ± 27.10
Glibenclamide	10 mg/kg	104.04 ± 5.26	103.32 ± 6.22	285.84 ± 9.83	204.84 ± 27.22
MEDR	250 mg/kg	102.24 ± 8.35	102.60 ± 3.27	288.36 ± 11.59	247.32 ± 19.98
MEDR	500 mg/kg	95.04 ± 8.00	105.84 ± 5.44	256.68 ± 27.63	196.20 ± 26.55

Group	Dose	Day 10	Day 14	Day 17	Day 21
Control	10 mL/kg	97.20 ± 5.94	105.12 ± 5.48	109.44 ± 5.50	102.60 ± 2.67
Diabetic Control	10 mg/kg	311.04 ± 21.11	318.60 ± 21.75	282.24 ± 30.41	243.72 ± 19.91
Glibenclamide	10 mg/kg	180.00 ± 13.31	168.12 ± 7.82	160.20 ± 21.35	146.52 ± 10.29
MEDR	250 mg/kg	176.76 ± 7.36	159.12 ± 11.34	141.48 ± 12.36	87.12 ± 6.65*
MEDR	500 mg/kg	181.80 ± 3.86	144.36 ± 13.67	119.16 ± 13.89	83.52 ± 7.86*

*Note:* Values were expressed as mean ± SEM (*𝑛* = 4). *p < 0.05, when compared with the control group (ANOVA followed by post hoc test).

### 3.5 In Silico Result

**FIGURE 5:**
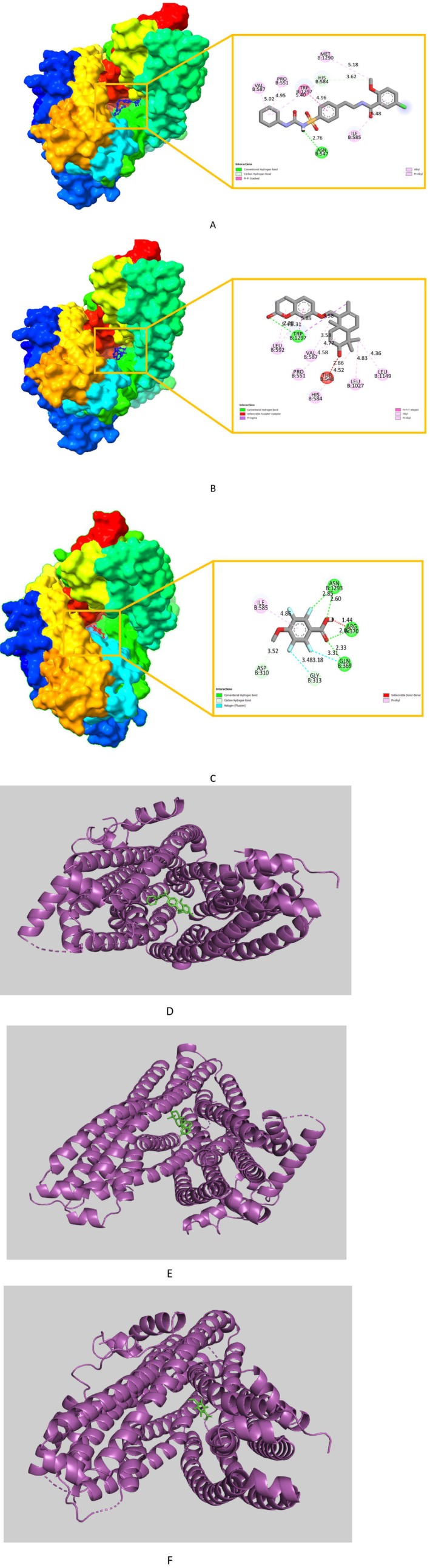
3D and 2D graphical visualization of Cox‐2 (5KIR) receptor with (A) Diclofenac, (B) Feselol, (C) 4‐methoxybenzoic acid, methyl ester, 3D view of Cox‐2 (5KIR) receptor with (D) Diclofenac, (E) Feselol, (F) 4‐methoxybenzoic acid, methyl ester.

**FIGURE 6:**
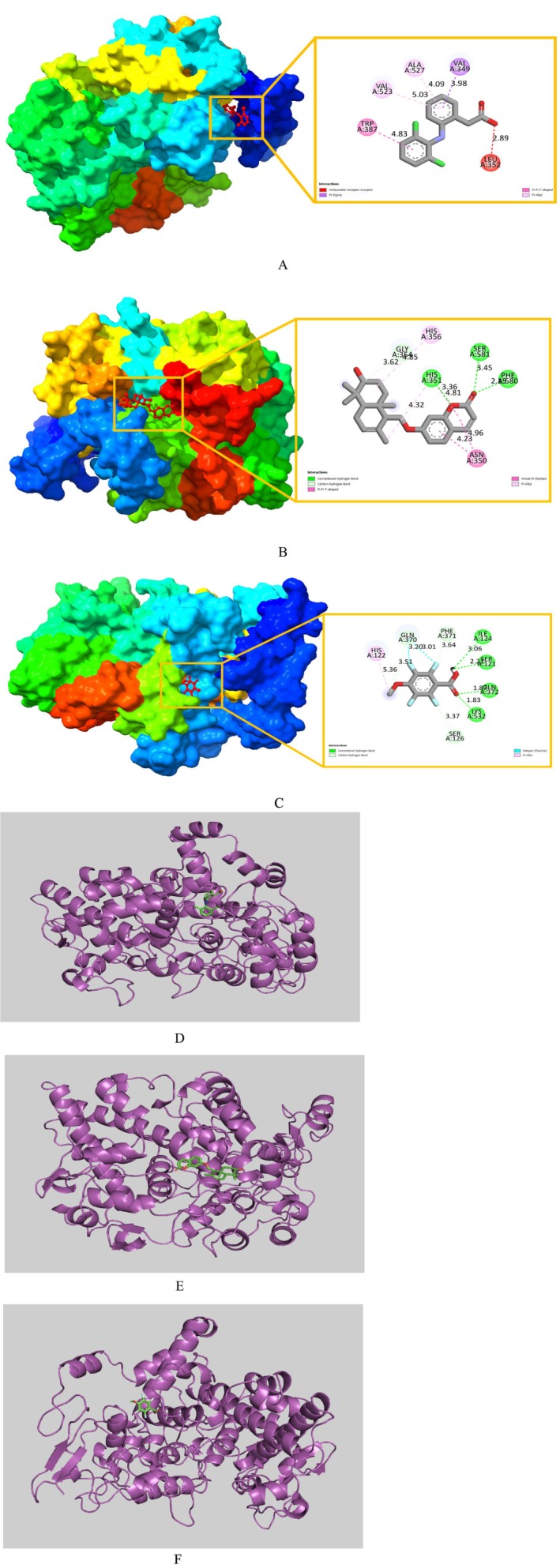
3D and 2D graphical visualization of SUR‐1 (6JB3) receptor with (A) Glibenclamide, (B) Feselol, (C) 4‐methoxybenzoic acid, methyl ester, 3D view of Cox‐2 (5KIR) receptor with (D) Glibenclamide, (E) Feselol, (F) 4‐methoxybenzoic acid, methyl ester.

The analgesic potential of COX2 (5KIR) receptor and hypoglycemic potential of SUR‐1 (6JB3) were studied using molecular docking simulations. Tables 6 and 7 summarize the docking scores. The reference drugs, Diclofenac sodium and Glibenclamide, had lower docking scores of −6.4 and −8.5 kcal/mol, respectively. Figures 5 and 6 represent the docking visualization of the top‐scored compounds. Table 8 shows drug‐like properties and computed ADMET properties of the top‐scored compounds of the docking result.

**TABLE 6:** Molecular docking results against COX‐2 (5KIR).

Sl. No.	Compounds	Docking Score (kcal/mol)	CAS No.
Standard	Diclofenac	−6.4	15307‐86‐5
1	4‐methoxybenzoic acid, methyl ester	−8.3	121‐98‐2
2	Feselol	−8.1	51020‐36‐1
3	beta‐Sitosterol acetate	−7.7	915‐05‐9
4	Retinal	−7.5	116‐31‐4
5	Naphthalenedione	−7.3	312924‐53‐1
6	alpha‐Tocopheryl acetate	−6.8	58‐95‐7
7	2,6‐Dihydroxybenzoic acid	−6.2	303‐07‐1
8	Propiophenone	−6.1	93‐55‐0
9	4‐Hydroxybenzoic acid	−6.0	99‐96‐7
10	Methyl beta‐D‐glucopyranoside	−5.9	709‐50‐2
11	3‐Methylbenzyl alcohol	−5.8	587‐03‐1
12	3‐Methylsalicylic acid	−5.7	83‐40‐9
13	Neophytadiene	−5.4	504‐96‐1
14	D‐Verbenone	−5.4	18309‐32‐5
15	Pinocarveol	−5.4	5947‐36‐4

**TABLE 7:** Molecular docking results against SUR‐1 (6JB3).

Sl. No.	Compounds	Docking Score (kcal/mol)	CAS No.
Standard	Glibenclamide	−8.5	10238‐21‐8
1	4‐methoxybenzoic acid, methyl ester	−9.0	121‐98‐2
2	Feselol	−8.4	51020‐36‐1
3	*β*‐Sitosterol acetate	−7.7	915‐05‐9
4	Naphthalenedione	−7.3	312924‐53‐1
5	Retinal	−6.8	116‐31‐4
6	α‐Tocopheryl acetate	−6.7	58‐95‐7
7	4‐methoxycinnamic acid, methyl ester	−6.2	3901‐07‐3
8	Vanillic Acid	−6.1	121‐34‐6
9	Propiophenone	−6.1	93‐55‐0
10	4‐Hydroxybenzoic acid	−6.0	99‐96‐7

**TABLE 8:** Drug‐like properties and computed ADMET properties of the top‐scored compounds.

Parameters	Feselol	4‐methoxybenzoic acid, methyl ester	Beta‐Sitosterol acetate	Interpretation
Molecular weight (g/mol)	382.5	166.17	456.755	
Octanol–water partition coefficient (log P)	4.941	4.39	8.596	
Hydrogen bond acceptors (HBA)	4	7	2	
Hydrogen bond donors (HBD)	1	0	0	
Number of rotatable bonds (RB)	3	9	8	
Topological polar surface area (TPSA)	59.67	83.68	26.3	
**Absorption**				
CaCO_2_ permeability log (cm/s)	−4.83	−4.67	−5.07	Highly permeable (> 0.90)
Human intestinal absorption	Absorbed	Absorbed	Absorbed	
Skin Permeability log Kp (cm/h)	−2.30	−2.41	−2.52	Low permeability (> −2.5)
P‐glycoprotein Inhibitor	Inhibitor	Inhibitor	Non‐Inhibitor	
P‐glycoprotein substrate	Non‐Substrate	Non‐Substrate	Non‐Substrate	
**Distribution**				
VDss	1.99	1.07	1.47	Low VDss (<−0.15) High VDss (> 0.45)
Plasma Protein Binding	92.38	89.0	87.73	Proper value (< 90%) Poor value (> 90%)
Fraction unbound	1.31	1.85	2.02	Numeric (Fu)
BBB permeability	Penetrable	Penetrable	Penetrable	
CNS permeability (log PS)	−2.17	−2.77	−2.12	Permeable (> −2) Not permeable (< −3)
**Metabolism**				
CYP1A2 inhibitor	Inhibitor	Inhibitor	Non‐Inhibitor	
CYP1A2 substrate	Non‐Substrate	Non‐Substrate	Non‐Substrate	
CYP2C19 inhibitor	Inhibitor	Inhibitor	Non‐Inhibitor	
CYP2C19 substrate	Substrate	Non‐Substrate	Substrate	
CYP2C9 inhibitor	Inhibitor	Inhibitor	Non‐Inhibitor	
CYP2C9 substrate	Non‐Substrate	Substrate	Non‐Substrate	
CYP2D6 inhibitor	Non‐Inhibitor	Non‐Inhibitor	Non‐Inhibitor	
CYP2D6 substrate	Substrate	Non‐Substrate	Non‐Substrate	
CYP3A4 inhibitor	Non‐Inhibitor	Inhibitor	Non‐Inhibitor	
CYP3A4 substrate	Non‐Substrate	Substrate	Substrate	
OATP1B1 inhibitor	Non‐Inhibitor	Non‐Inhibitor	Non‐Inhibitor	
OATP1B3 inhibitor	Non‐Inhibitor	Non‐Inhibitor	Non‐Inhibitor	
**Excretion**				
Total Clearance log (mL/min/kg)	6.14	2.92	7.08	
Renal OCT2 inhibitor	Non‐Inhibitor	Non‐ Inhibitor	Non‐Inhibitor	
Excretion Half‐Life (hours)	Half‐Life < 3hs	Half‐Life < 3hs	Half‐Life < 3hs	
AMES toxicity	Toxic	Safe	Safe	
Carcinogenesis	Safe	Safe	Safe	
Maximum tolerated dose (human) log (mg/kg/day)	0.17	1.14	1.68	Low (≤ 0.477) High (> 0.477)
hERG Blockers	Toxic	Safe	Toxic	
Oral rat acute toxicity (mol/kg)	2.41	2.7	2.05	
Oral rat chronic toxicity (log(mg/kg/b.w./day))	1.5	1.35	1.85	
Liver Injury I (DILI)	Safe	Toxic	Toxic	
Liver Injury II	Toxic	Toxic	Toxic	
Skin sensitization	Toxic	Safe	Toxic	

## 4 Discussion

This study provides the first scientific evidence of the analgesic and hypoglycemic properties of *D. racemosus* , suggesting its potential as a novel candidate for the management of pain and metabolic disorders, although no prior traditional use has been reported. ADMET and *in silico* molecular docking studies supported the methanolic extract's (MEDR) significant pharmacological profiles, indicating that its phytoconstituents might be valuable lead compounds for drug development.

The observed inhibition of acetic acid‐induced writhing, formalin‐induced paw licking, and hot plate response indicates that MEDR has both peripheral and central analgesic potential. Pain modulation includes both peripheral and central pathways. The extract effectively reduced prostaglandin production in the acetic acid‐induced writhing test, showing a dose‐dependent decrease in writhing motions with 66.45% inhibition at 500 mg/kg. These results are similar to those found for other medicinal plants, including *Quassia amara* and *Amaranthus blitum*, which have analgesic qualities (Jahan et al. 2022).

The extract showed significant diminution in both the early (neurogenic) and late (inflammatory) phases of the formalin test, indicating that MEDR disrupts inflammatory mediators such as bradykinin and prostaglandins. Additionally, the extended latency period, which neared the activity of morphine at higher dosages, demonstrated the hot plate test's confirmation of its central analgesic impact. Together, these results suggest that MEDR's analgesic effects could be mediated via central pain circuit regulation and suppression of COX‐2‐driven prostaglandin production.

GC–MS analysis revealed the presence of bioactive compounds such as Feselol, 4‐methoxybenzoic acid methyl ester, and *β*‐sitosterol acetate, all of which are known to possess anti‐inflammatory or analgesic potential. Similar studies for the *Sapindaceae* family for analgesic properties include similar findings about the aforementioned compounds, which validate the current findings (Olayinka et al. 2021; Shah et al. 2026). In silico docking supported this finding, as 4‐methoxybenzoic acid methyl ester exhibited a higher binding affinity to COX‐2 (−8.3 kcal/mol) than diclofenac (−6.4 kcal/mol), forming stable hydrogen bonds with key residues at the active site. Similar interactions were also observed for β‐sitosterol acetate and 4‐methoxybenzoic acid derivatives, confirming their possible role in modulating cyclooxygenase activity.

The extract reduced fasting blood glucose in a dose‐dependent manner; on the 21st day of therapy, 500 mg/kg was as effective as the common medication Glibenclamide. Since STZ at 60 mg/kg induces a Type 1 diabetic model through selective pancreatic β‐cell destruction, the observed antihyperglycaemic effect is more likely associated with protective effects on residual β‐cells, attenuation of pancreatic damage, or preservation of endogenous insulin activity, rather than improvement of insulin resistance. Similar mechanisms have been demonstrated for other Anacardiaceae species, such as *Mangifera indica* and *Anacardium occidentale* (Ngo et al. 2019). The methyl ester of 4‐methoxybenzoic acid showed the highest binding affinity (−9.0 kcal/mol) to the sulfonylurea receptor‐1 (SUR‐1), exceeding that of Glibenclamide (−8.5 kcal/mol). Although molecular docking showed strong binding affinity of 4‐methoxybenzoic acid methyl ester to the sulfonylurea receptor‐1 (SUR‐1), suggesting a possible role in modulating pancreatic K‐ATP channels, direct stimulation of insulin secretion cannot be conclusively established in the absence of insulin measurement. Feselol and *β‐*sitosterol acetate both have shown an insignificant affinity for SUR‐1, indicating that a variety of phytochemicals may contribute to the extract's hypoglycemic effects. The ADMET profile of high‐scoring compounds expressed significant drug‐like properties. Feselol and 4‐methoxybenzoic acid methyl ester both showed good plasma protein binding, blood–brain barrier permeability, and strong gastrointestinal absorption. Additionally, their non‐carcinogenic and non‐mutagenic properties show that they are safe for further study. Feselol has a low clearance rate and expected pharmacokinetic stability, which may allow for structural modification to optimize it despite its slight hepatotoxic risk.

## 5 Conclusion

The combined in silico and in vivo data highlight *the medicinal promise of D. racemosus leaves* as a source of bioactive compounds for diabetes and pain management. The dependability of the study results is strengthened by the correspondence between the computational and pharmacological results. The biological activities identified are further supported by the presence of terpenoids and phenolic compounds, as determined by GC–MS. The antihyperglycemic activity observed in the STZ‐induced Type 1 diabetic model suggests possible pancreatic protective effects and maintenance of glucose homeostasis. Although the study presents robust data, further biochemical and mechanistic investigations are necessary to elucidate the exact molecular pathways underlying these activities. Isolation and characterization of the major bioactive compounds, followed by enzyme inhibition assays, histopathological evaluation of pancreatic tissue, insulin level estimation, receptor‐binding studies, and chronic toxicity evaluation, would provide deeper insight. Moreover, future research employing molecular dynamics simulations could validate the stability of ligand–protein interactions observed in docking.

## Author Contributions

**Nilufar Sultana:** conceptualization, methodology, writing – review and editing, writing – original draft, supervision, project administration. **Sanjana Haque Esha:** investigation, data curation. **Md. Mehedi Hasan:** investigation, formal analysis, software. **Abdullah Ripon:** writing – review and editing, data curation. **M. A. Rafi:** investigation, software, data curation. **Md. Rakibul Islam:** investigation, software, data curation. **Payar Hossain:** methodology, investigation. **Md Sajib Ali:** software, formal analysis, investigation, visualization, data curation.

## Funding

The authors have nothing to report.

## Ethics Statement

The Institutional Animal Ethics Committee of the Department of Pharmacy, Faculty of Science, Engineering, and Technology, Manarat International University, Bangladesh, authorized the animal experiments used in this study. All authors declare that the “Principles of Laboratory Animal Care” (NIH publication No. 85–23, revised 1985) and any relevant national regulations were followed. All experiments, including the investigation of the plant under study, were reviewed and approved by Manarat International University's ethical committee (approval number MIU/SEST/ERC/2025/001).

## Conflicts of Interest

The authors declare no conflicts of interest.

## Data Availability

The data that support the findings of this study are available from the corresponding author upon reasonable request.
